# 

**Published:** 1889-06-29

**Authors:** 


No. 144, Vol. VI.—June 29th, 1889.
[Registered at the General Post Office as a Newspaper.]

Monthly Parts, 6d.; post free 7½d.

Ditto per Annum, 7s. 6d., post free.

